# Immunological Activity and Gut Microbiota Modulation of Pectin from Kiwano (*Cucumis metuliferus*) Peels

**DOI:** 10.3390/foods11111632

**Published:** 2022-05-31

**Authors:** Minqian Zhu, Ya Song, M. Carmen Martínez-Cuesta, Carmen Peláez, Enru Li, Teresa Requena, Hong Wang, Yuanming Sun

**Affiliations:** 1 Guangdong Provincial Key Laboratory of Food Quality and Safety, College of Food Science, South China Agricultural University, Guangzhou 510642, China; zhuminqian@stu.scau.edu.cn (M.Z.); songya_1990@163.com (Y.S.); 19120536638@163.com (E.L.); ymsun@scau.edu.cn (Y.S.); 2 Guangdong Laboratory for Lingnan Modern Agriculture, College of Food Science, South China Agricultural University, Guangzhou 510642, China; 3 Department of Food Biotechnology and Microbiology, CIAL-CSIC, 28049 Madrid, Spain; carmen.martinez@csic.es (M.C.M.-C.); carmen.pelaez@csic.es (C.P.)

**Keywords:** kiwano, *Cucumis metuliferus*, pectin, immune-enhancing, microbiota simulation

## Abstract

For developing the recycling of fruit by-products from kiwano, a polysaccharide was extracted from kiwano (*Cucumis metuliferus*) peels, namely *Cucumis metuliferus* peels polysaccharide (CMPP), with the aim of investigating the potential beneficial effects. The composition of polysaccharides was analyzed by chemical methods. RAW264.7 macrophages cells and the microbiota dynamics simulator (BFBL gut model) were used for in vitro study. The result showed that CMPP mainly consists of glucuronic acid, arabinose, galactose and rhamnose. By intervening with RAW264.7 cells, CMPP promoted cell proliferation and showed immune-enhancing activity, which significantly (*p* < 0.05) induced the release of nitric oxide (NO), tumor necrosis factor α (TNF-α) and interleukin 6 (IL-6) at a concentration of 50 μg/mL. In addition, CMPP had an impact on the composition of the gut bacteria, increasing the growth of *Akkermansia*, *Bacteroides*, *Bifidobacterium*, *Feacalibacterium*, and *Roseburia*. During the intake period, acetic, butyric and propionic acids were all increased, especially (*p* < 0.05) in the descending colon. Moreover, a decrease in ammonia concentration (10.17 ± 0.50 mM in the ascending colon, 13.21 ± 1.54 mM in the transverse colon and 13.62 ± 0.45 mM in the descending colon, respectively) was observed. In summary, CMPP can be considered as a pectin, showed immunological activity and function of gut microbiota modulation. This study could be the scientific basis of developing kiwano peels as beneficial to human health.

## 1. Introduction

The gastrointestinal tract contains the human body’s largest immune organ, as well as an enormous number of microorganisms. The microorganisms played a critical role in the host homeostasis, like producing metabolites, resisting viral and pathogenic infections, and promoting immune system maturation [1,2]. Providing non-digestible compounds can bring beneficial physiological effects on the host due to gut microbiota modulated metabolites; some of these compounds are referred to as prebiotics [3]. One key mechanism of prebiotic benefits is that the intestinal microbiota produces short-chain fatty acids (SCFAs) from non-digestible carbohydrates, which can reduce intestinal pH to exclude pathogens and have antimicrobial activity [4]. Prebiotic food has an important role in health well-being, especially for treating overweight or obesity by modulating gut microbiota to improve metabolism.

Kiwano (*Cucumis metuliferus*), also referred to as African cucumber, horned melon and jelly melon, belongs to *Cucurbitaceae*. It is naturally distributed in the tropical and subtropical sub-Saharan regions of Africa, and is also commercially produced in Kenya, New Zealand, France, Israel, Australia, China, etc. [5]. Kiwano fruit has been reported for antimicrobial [6], antifungal [7], antiviral [8], anti-ulcer [9] and hypoglycemic [10] activities. The weight of the peel accounts for about a quarter of the whole kiwano, which is a by-product after juicing. It is reported that the composition of kiwano peel is carbohydrate (54.84%), moisture (18.40%), fiber (11.34%), fat (8.89%), ash (3.59%), and protein (2.95%). The peel also contains alkaloids, flavonoids, saponins, tannins, glycosides, terpenoids and phenols [11]. From the results of the composition analysis, the polysaccharide is the largest proportion in the kiwano peel. In general, the cell wall of the peel is rich in pectin, which was composed of galacturonic acid (Gal A), galacturonic acid (Gal A), glucose (Glu), arabinose (Ara), galacturonic (Gal) and rhamnose (Rha), etc [12]. The composition and structure of pectin are related to function, including antioxidant capacity, immunological activity and in vitro anti-cancer activity. Besides, pectin from different sources exerts distinct effects on the gut microbiota. For example, citrus pectin increased the abundance of *Bacteroidetes* in the rat gut, while apple pectin decreased the abundance of *Bacteroides* and increased the population of *Clostridiales* [13]. Various properties of pectin show high biological activity; however, the pectin from *C. metuliferus* peels (CMPP) is generally discarded and overlooked. Therefore, it is considered worthy of further study.

Research in animal and human trials has many disadvantages, including ethical constraints, high costs, individual differences and limitations in sampling. In vitro models can better control the experimental conditions [14]. However, in vitro investigations have limitations regarding applicability, and more effects of function should be considered [14]. In this study, composition of CMPP was elucidated by monosaccharide analysis and ultraviolet and visible (UV) spectrum. Based on this, the cell viability and immune factors of mouse mononuclear macrophage leukemia cells (RAW264.7) were used for evaluating the immune activity on CMPP, such as nitric oxide (NO), tumor necrosis factor α (TNF-α) and interleukin 6 (IL-6). In addition, the functional biology of the lactic bacteria (BFBL) gut model is a dynamic simulator of the entire human gut in which environmental conditions (e.g., pH, temperature, anaerobiosis and residence time) are controlled to resemble in vivo conditions [15]. This model was used to evaluate changes in the human gut microbial community structure and ammonium and SCFAs production.

## 2. Materials and Methods

### 2.1. Materials and Reagents

The fresh yellow kiwanos were selected for a diameter of 12 ± 1 cm, width of 6 ± 1 cm, with hard thorns. They were obtained from local markets in Fujian province, China. After separating and washing manually, the peels were kept at −80 °C for 48 h and then freeze-dried. The lyophilized peels were ground in a blender and passed through the 0.5 mm sieve. Then, the peel power was stored in new plastic sample bags at −80 °C until analyses.

Standard monosaccharides, trifluoroacetic acid (chromatographic grade), mucin (porcine stomach), apple pectin, and arabinogalactan were purchased from Sigma-Aldrich (Burlington, MA, USA). Potato starch and glucose were purchased from BD, Difco™ (Thermo Fisher Scientific, Waltham, MA, USA). Yeast extract and peptone were purchased from Oxoid, Thermo Scientific (Waltham, MA, USA). Xylan was a product of Carbosynth (Compton, UK). L-cysteine was a product of Panreac, AppliChem (Darmstadt, Germany). The other reagents used were of analytical grade. Macrophage RAW264.7 was obtained from the American Type Culture Collection (ATCC, Manassas, VA, USA). Dulbecco’s modified Eagle medium (DMEM), fetal bovine serum (FBS) penicillin streptomycin and trypsin were purchased from Gibco BRL (Gaithersburg, MD, USA). The nitric oxide detection kit was a product of the Beyotime Institute of Biotechnology (Shanghai, China). The enzyme-linked immunosorbent assay (ELISA) kits, including TNF-α and IL-6, were products of NeoBioscience Biotechnology Co. LTD (Shenzhen, China). The fecal slurry was from healthy volunteers who received no antibiotics within the prior 3 months. The study was approved (4 July 2016) by the Clinical Ethics Committee of Hospital Ramón y Cajal with the code 394/14 and Spanish Council of Scientific Research (CSIC).

### 2.2. Pectin Extraction

The CMPP was prepared in the same way that is referred to in the previous study [16]. The dried fruit powder was dipped into distilled water at the ratio of 1:30 (*w*/*v*) and extracted twice at 70 °C for 3 h. The two extracts were combined, and centrifuged at 12000 rpm for 15 min to remove precipitated compounds. After removing protein by 4 vols of Sevag reagent (CHCl_3_: n-C_4_H_9_OH = 4:1, *v*/*v*), the supernatant was precipitated with 95% ethanol. The residue was centrifugated at 12000 rpm for 15 min, then dissolved in distilled water, dialyzed in a cellulose membrane (Mw 5000 Da) against distilled water for 3 days, and followed by lyophilization at −80 °C for 48 h to obtain the CMPP.

### 2.3. Determination of Chemical Composition

Using sodium nitrite–aluminum nitrate determined the total flavonoids in CMPP [17]. The Folin–Ciocalteau method was used to detect the total phenols [18]. A DU-8000 spectrophotometer (NanoDrop 2000c, Thermo Fisher Scientific, MMAS, Waltham, MA, USA) was checked for nucleic acids and proteins in the polysaccharides solution in the range of 190 to 800 nm [19].

### 2.4. Monosaccharide Analysis

Weighing about 5 mg of CMPP was hydrolyzed with 1 mL 2.5 mol/L trifluoroacetic acid (TFA) at 121 °C for 2 h, and the TFA was removed three times with methanol and nitrogen gas. Then, the dissolved hydrolytic product was injected into high-performance anion-exchange chromatography with pulsed amperometric detection (HPAEC-PAD). The equipment consisted of the ICS-5000 system (Thermo Fisher Scientific, Waltham, MA, USA) with Dionex™ CarboPac™ PA20 (150 mm × 3 mm, 6.5 μm) and a pulsed amperometric detector. The work of quantification was operated by professional staff from San-Shu Biotech Co. (Shanghai, China).

### 2.5. Cell Viability Assay

Incubation conditions for macrophage RAW264.7 cells were the incubator with 5% CO_2_ in humidified atmosphere at 37 °C. The medium was 1% DMEM with 10% heat-inactivated FBS, penicillin and streptomycin. Cell viability was estimated by the 3-(4, 5-dimethylthiazole-2-yl)-2, 5-bromodiphenyltetrazolium (MTT) method. Macrophage RAW264.7 cells were cultured in a 96-well plate at a concentration of 3 × 10^4^ cells/well, and polysaccharides were added at a concentration of 0–50 µg/mL. After 24 h, 10 μL of MTT solution with a concentration of 5 mg/mL was added to the 96-well plate. After incubating for 4 h of reaction, the supernatant was removed, adding 100 μL of dimethyl sulfoxide (DMSO) to dissolve the crystals. Absorbance was recorded at the UV wavelength of 490 nm.

### 2.6. Detection of NO and Cytokines

The method of detecting NO and cytokine (TNF-α and IL-6) production followed the description from previous study [20]. RAW264.7 cells were cultured in a 24-well plate (5 × 10^5^ cells/100 L) with 0–50 µg/mL polysaccharide and lipopolysaccharide (LPS, positive group), respectively, for 24 h. Griess reagent was used to detect NO in the culture supernatant, and cytokines were detected by ELISA. The absorbance was recorded at 540 nm in the UV spectrum.

### 2.7. Colon Microbiota Dynamic Simulation

The BFBL dynamic gastrointestinal simulator is a reactor that simulates the human gut, simulating the small intestine, ascending colon (AC), transverse colon (TC) and descending colon (DC) [15]. The nutritive medium was filled and pre-conditioned in AC (250 mL), TC (400 mL) and DC (300 mL). These colon reactors were inoculated and equilibrated overnight at 37 °C filled with nitrogen. Then, 0.5 M NaOH and 0.5 M HCl were used to maintain pH values of 5.7 ± 0.2 in AC, 6.3 ± 0.2 in TC and 6.8 ± 0.2 in DC, respectively.

Nutritive medium contained mucin from porcine stomach (4 g/L), potato starch (3 g/L), yeast extract (3 g/L), apple pectin (2 g/L), arabinogalactan (1 g/L), peptone (1 g/L), xylan (1 g/L), L-cysteine (0.5 g/L) and glucose (0.4 g/L), and all of the ingredients were dissolved in 1000 mL distilled water, adjusting pH at 2.0 and sterilizing at 121 °C for 21 min. Fecal slurry was inoculated to the BFBL model. Additionally, fecal samples were diluted (20% *w*/*w*) in 0.1 mol/L sterile phosphate buffered saline with 1 g/L sodium thioglycolate at pH 7.0.

To simulate gut microbial communities under steady state conditions, the BFBL models were fed 75 mL nutrient medium with 40 mL (12 g/L NaHCO_3_, 6 g/L oxgall dehydrated fresh bile and 0.9 g/L porcine pancreatine) at pH 2.0, three times per day [21]. The medium was digested at 37 °C for 2 h, and the digestion was delivered to the colon compartment at 5 mL/min. All the compartments were flushed with nitrogen during the feeding process to maintain the anaerobic conditions. After the stabilization period, the medium supplemented with CMPP (3.5 g/L) was fed to the BFBL model for seven days (intake period). A one-week washout period followed. Throughout the experiment, samples were collected daily from the three colonic compartments and centrifuged at 10,000 rpm for 10 min at 4 °C. The supernatant was stored at −20 °C until analysis for SCFAs and ammonium; the pellet samples were stored at −80 °C until analysis for gut microbiota composition and quantification by quantitative real-time polymerase chain reaction (qPCR).

### 2.8. Microbiological Analysis

Extraction of DNA from pellet samples followed protocol Q for non-commercial IHMS, suggested by the International Human Microbiome Consortium [22]. Standards, primers, amplicon size, and annealing temperature for total bacteria, *Akkermansia*, *Bacteroides*, *Bifidobacterium*, *Feacalibacterium* and *Roseburia* were described previously [23]. DNA samples and standards were quantified by qPCR with the SYBR^®^ green method in a ViiA7 Real-Time PCR System (Life Technologies, Carlsbad, CA, USA).

### 2.9. SCFAs Analysis

The supernatant samples were filtered and 0.2 μL was injected into a high efficiency liquid chromatography (HPLC) system (Jasco, Tokyo, Japan) equipped with a UV-975 detector and automatic injector. The SCFAs were separated on a Rezex ROA Organic Acids column (300 × 7.8 mm) (Phenomenex, Macclesfield, UK) with a thermostat at 50 °C. The mobile phase was a linear gradient of 0.005 M sulfuric acid in HPLC-grade water at 0.6 mL/min, and the elution curves were monitored at 210 nm. A calibration curve for SCFA was established in the concentration range of 1–100 mM.

### 2.10. Ammonium Determination

Ammonium was determined by Nessler’s reagent (Sigma) [24]. A calibration curve was prepared using ammonium chloride in the 0–25 mM range. the absorbance was recorded at a UV wavelength of 425 nm, and analyses of samples were performed in duplicate.

### 2.11. Statistical Analysis

**Figure 1:**
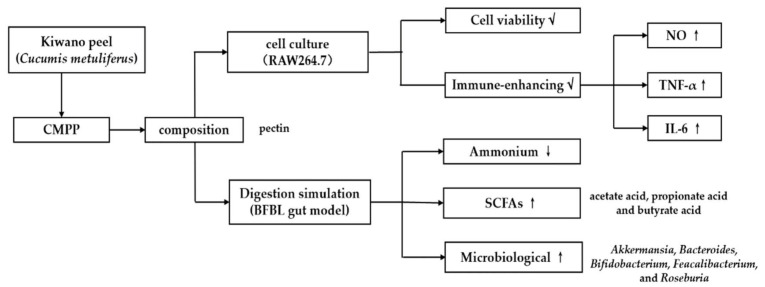
Experimental flow diagram. The diagram shows sequence of experimentation done in the current investigation and what are various test/analysis done during the scheme of experimentation. Abbreviation presented as: Cucumis metuliferus peels polysaccharide (CMPP), mouse mononuclear macrophage leukemia cells (RAW264.7), functional biology of lactic bacteria (BFBL), short-chain fatty acids (SCFAs), nitric oxide (NO), tumor necrosis factor α (TNF-α) and interleukin 6 (IL-6).

Experiments were repeated three times, and the data were expressed as mean ± standard deviation. Statistical analysis was performed by one-way ANOVA with SPSS 20.0 software (IBM Corporation, Armonk, NY, USA). Differences were considered significant when *p* < 0.05, and were highly significant at *p* < 0.01 (Figure 1).

## 3. Results and Discussion

### 3.1. Chemical Composition Analysis

**Figure 2:**
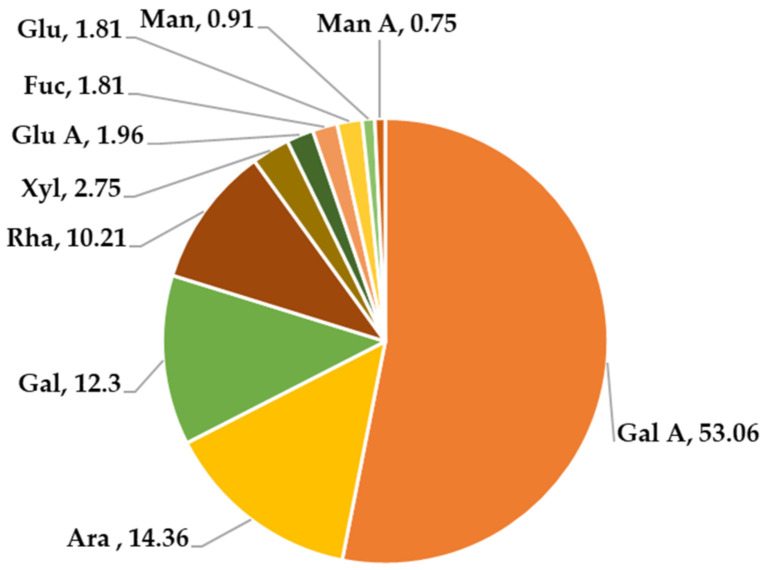
Monosaccharide composition of CMPP. The numbers of monosaccharide composition presented mole percentages. The colors are only used for distinction. Abbreviation presented as: galacturonic acid (Gal A), rhamnose (Rha), arabinose (Ara), galacturonic (Gal), glucose (Glu), xylose (Xyl), Fuc (fucose), glucose acid (Glu A), mannose acid (Man A) and mannose (Man).

CMPP was performed to decipher its main components by chemical methods. Flavones and polyphenols were not detected in the CMPP. Moreover, no absorbance peaks at 260 nm and 280 nm were observed in the UV spectrum (results not shown), indicating that nucleic acids and proteins were also not detected in CMPP. Using HPAEC-PAD detected the monosaccharide composition. As shown in Figure 2, CMPP was composed mainly of 10 monosaccharides, including galacturonic acid (Gal A), rhamnose (Rha), arabinose (Ara), galacturonic (Gal), glucose (Glu), xylose (Xyl), Fuc (fucose), glucose acid (Glu A), mannose acid (Man A) and mannose (Man). Compared with previous study, the monosaccharide composition of CMPP was similar with CMPP-1 and CMPP-2 [16]. Gal A was the main constituent with a molar percentage value of 53.06% in CMPP; Ara, Gal and Rha were the top three in neutral sugars. It is reported that the pectin from sweet cherry (*Prunus avium*) contained 49.38% of Gal A, of which Ara, Gal and Rha were also the top three monosaccharides [25]. In addition, the results of pectic from apple by-product [26] and *Morus alba L* showed similar results [27]. Therefore, the monosaccharide composition results indicated that CMPP was consistent with pectin.

### 3.2. Immune Activity Analysis

#### 3.2.1. Cell Viability

**Figure 3:**
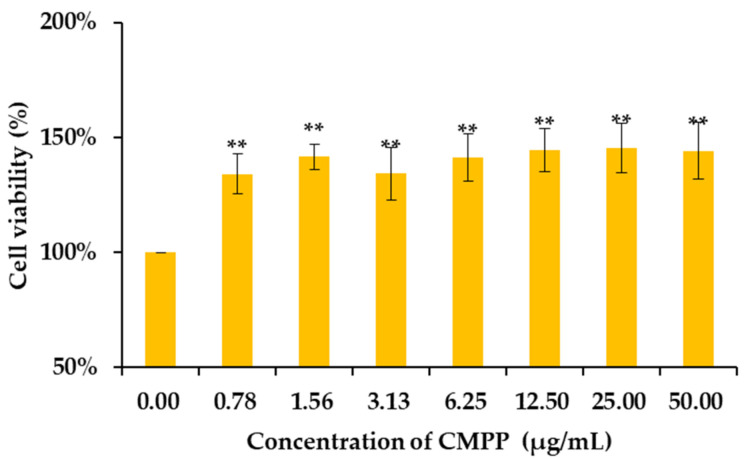
Effects of CMPP on cell viability of RAW264.7 macrophages. The value of cell viability over 100% means promoting cell growth. Data were presented as mean ± SD. ** were highly significantly different, *p* < 0.01, vs. the control group.

We used MTT crystal formation to assess the cell viability of CMPP on RAW264.7 macrophages, which was proportional to the number of cells [28]. Compared with the blank control, the cell viability of CMPP was higher than 100% at the concentration of 0.78–50 μg/mL for 24 h (Figure 3), remarkably to 134% ± 9% and 144% ± 12% (*p* < 0.01), respectively. The result indicated that CMPP was not cytotoxic and could promote cell proliferation on RAW264.7 at 0.78–50 μg/mL, which had a wider concentration range than CMPP-1 (0.78–3.13 µg/mL) and CMPP-2 (12.5–25 µg/mL) [16]. The effect was also consistent with the polysaccharides from coreopsis (*C. tinctoria*) [29], *Ficus carica* [28], herbal *Gynostemma pentaphyllum* tea [30] and *Dendrobium officinale* [31]. Therefore, this concentration range can be considered for subsequent studies.

#### 3.2.2. Immune-Enhancing Activity

**Figure 4:**
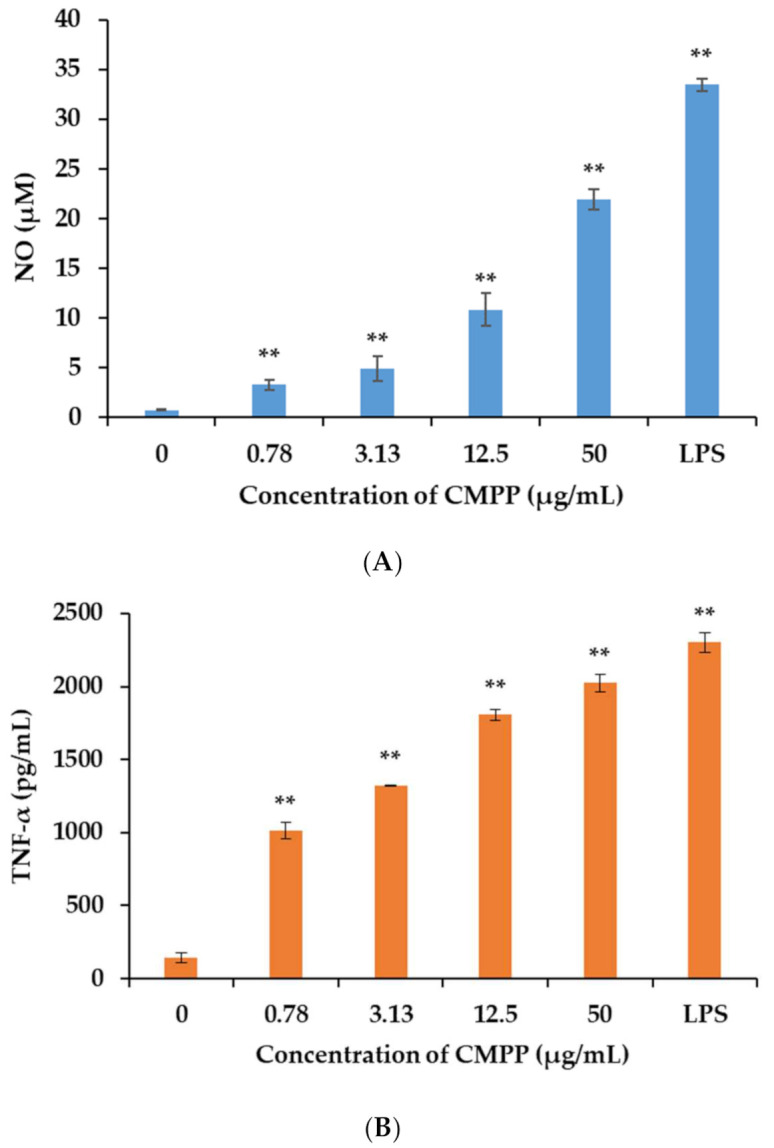

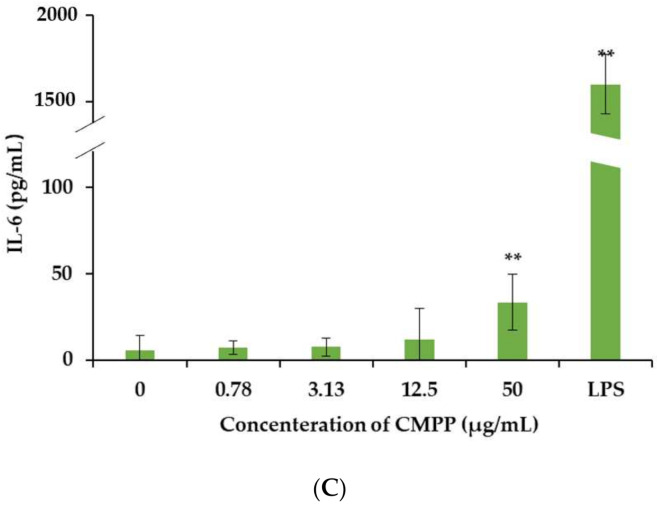
Production of NO (**A**), TNF-α (**B**) and IL-6 (**C**) in RAW264.7 cells activated by CMPP. LPS group was a positive group of overproduction, which means 0.78–50 µg/mL of CMPP was more moderate than LPS. Data were presented as mean ± SD. ** were highly significantly different, *p* < 0.01, vs. the control group (0 µg/mL).

The immune responses of macrophages were highly relevant to human health, and an increased secretion of cytokines (NO, IL-6, TNF-a) is often used to assess immunological activity [32]. As shown in Figure 4A, the treatment with CMPP at the concentration range of 0.78–50 μg/mL significantly (*p* < 0.01) increased NO production in a dose-dependent manner. A release of TNF-α (Figure 4B) also increased significantly (*p* < 0.01) with the concentration of CMPP in a dose-dependent manner. The IL-6 secretion (Figure 4C) of RAW264.7 increased significantly (*p* < 0.01) when treating with 50 µg/mL of CMPP. At the highest concentration of CMPP (50 µg/mL), NO, TNF-α and IL-6 production were all inferior to the LPS group, which suggested a more moderate immune regulation of CMPP than LPS [33]. Comprehensively, CMPP had better immune-enhancing activity than CMPP-2 (6.25 µg/mL) at the equivalent concentration, and its effect was close to CMPP-1 (0.78 µg/mL) [16]. The immune-enhancing activity was described with pectin from sweet cherry and mulberry [25,27], which also effectively activated RAW264.7 cells to release NO and cytokines. These results indicated that CMPP had immunological activity.

### 3.3. Microbial Metabolite Analysis

**Figure 5:**
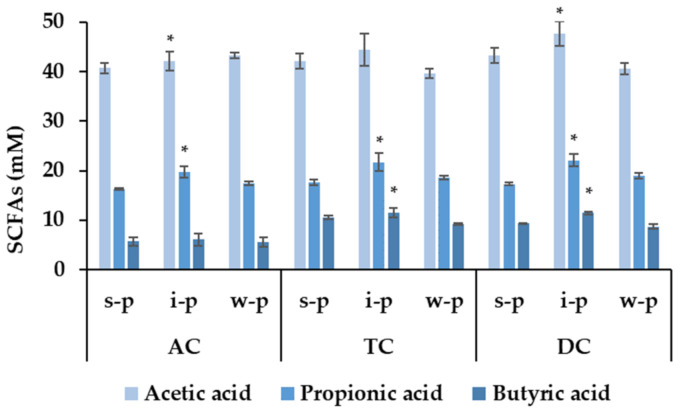
SCFAs content in the ascending colon (AC), transverse colon (TC) and descending colon (DC) of the stabilization period (s-p), intake period (i-p) and washout period (w-p). The colors are only used for distinction. Data were presented as mean ± SD. * was significantly different, *p* < 0.05, vs. the stabilization period.

The major metabolites of indigestible carbohydrates metabolism by the gut microbiota were the SCFAs. The results of SCFAs concentration of stabilization period, intake period and washout period in the AC, TC and DC compartments are shown in Figure 5. During the stabilization period, acetic acid ranked first detected in SCFAs content, with butyric acid and propionic acids behind. Compared with the stabilization period, the average increase of acetic acid content of AC, TC and DC was 1.07, 1.05 and 1.10-fold, respectively, during the intake period. Likewise, the propionic acid content of AC, TC and DC increased 1.26-, 1.21- and 1.24-fold, respectively; the content of butyric acid increased 1.07-, 1.10- and 1.22-fold, respectively. Acetic acid, butyric and propionic acids all increased, especially (*p* < 0.05) in DC during the intake period. Afterwards, the SCFAs concentrations decreased during the washout period, the data of which were similar to the stabilization period. The effect was consistent with the report of inulin feeding to a three-stage continuous culture system [34]. It is well known that SCFAs were important products of the fermentation of pectin polysaccharides. Acetic acid and butyric acid were produced by fermentation of galactose and galacturonic acid. Acetic acid is involved in lipid metabolism as the main energy source for muscles. Butyric acid is related to epithelial cell protection and has anti-inflammatory effects, while propionic acid is mostly the product of arabinose and glucose fermentation [35], which is related to the regulation of energy pathways and in the release of satiety peptides/hormones. Combining the results of the monosaccharide composition, CMPP was rich in Gal A, Ara, Gal and Rha, which can be fermented to increase the SCFAs. Propionic was significantly (*p* < 0.05) increased in all three compartments indicating that propionic bacteria producers can use CMPP. The main SCFAs derived from mango peel pectic oligosaccharide were acetic acid and propionic acid [36]. SCFAs content were also increased significantly in both isolated apple pectin and fruit mango pulp, with the isolated pectin favoring butyrate production, while propionate production was increased by the mango pulp [37]. Different dietary fibers have similar but not identical effects on SCFAs, requiring further study.

The content of ammonium was determined to evaluate the fermentation of proteins, of which metabolites or end products from proteolytic fermentation were potentially toxic. The result showed the ammonium content in stabilization period reached average values (±SD) of 12.58 ± 2.34 mM in AC, 15.65 ± 2.34 mM in TC and 14.32 ± 0.74 mM in DC, respectively. With the CMPP, the content of ammonium decreased in the three reactors (10.17 ± 0.50 mM in AC, 13.21 ± 1.54 mM in TC and 13.62 ± 0.45 mM in DC, respectively). Compared with the intake period, the content of ammonium increased in the washout period, which was 10.28 ± 1.35 mM in AC, 14.24 ± 1.50 mM in TC and 13.98 ± 0.97 mM in DC, respectively. These results showed that proteolytic metabolism diminished during the CMPP intake period, similar with citrus pectin in vitro gastrointestinal simulation [38]. Likewise, the ammonium production decreased gradually with the fermentation of arabinoxylans in a gut microbiota simulator [39]. A decrease of protein fermentation in the large intestine when humans and animal models were supplemented with non-digestible carbohydrates, which was consistent with reducing the genotoxicity of fecal water [40].

The potential prebiotic effect of CMPP was also analyzed by the changes of representative gut microbiota in the BFBL gut model. Based on this, average bacterial counts of AC, TC, and DC were detected by qPCR in the last three days of the three experimental periods (Table 1). Compared to the stabilization period, the number of *Bacteroides*, *Feacalibacterium*, and *Roseburia* increased after the CMPP intake period. Although the data of *Akkermansia* in AC were below the detection level, there was an increased tendency in TC. *Akkermansia* could use the galactose and glucose from CMPP as a carbon source to produce propionic acid. It is reported that *Akkermansia* was related to the improvement of inflammatory diseases [41] and protection from metabolic diseases, including prevention of type 2 diabetes (T2D) and reduction of cardiovascular disease [42]. *Bacteroides* increased during the test period, mainly in AC (*p* < 0.05), which is related to production of propionic and acetic acids. The genera *Akkermansia*, *Bacteroides*, *Feacalibacterium* and *Roseburia* were depleted in T2D Chinese patients [43]. On the other hand, *Bifidobacterium* had a slight tendency to increase during the test period, but not significantly. This genus can degrade a variety of carbon sources, including monosaccharides and disaccharides, and complex carbohydrates such as xylose, galactose and oligofructose [44]. *Bifidobacterium* produced acetic acid and lactic acid by fermenting carbohydrates, and through a cross-feeding mechanism, it may stimulate the production of butyric acid [45]. The proportion of *Bifidobacterium* was shown to improve diabetes-related complications and obesity [41]. CMPP also showed a tendency for increasing *Feacalibacterium* in TC and DC (*p* < 0.05). Compared with chronic pancreatitis patients and the obese group, *Feacalibacterium* was higher in the control group, which was also positively correlated with inflammation improvement [46]. *Roseburia* was increased with treatment of CMPP, especially in AC (*p* < 0.05). It can ferment several carbohydrates, including xylose, galactose, glucose, sucrose, starch and glycogen [47]. Studies have shown that healthy people had higher abundance of *Roseburia* than patients with T2D [48]. *Feacalibacterium* and *Roseburia* were recognized key producers of butyrate [49], and their reduction was labeled as predicting diabetes in European women [43]. Overall, the bacterial groups that increased during the intake period were mainly related to T2D, obesity and reducing inflammation, which suggested CMPP could improve these symptoms. These results were similar to the dynamic in vitro digestion of citrus [38]. A high increase in SCFAs, especially the concentration of acetate and butyrate, and a decrease in ammonia concentration were also detected after feeding pectin; these changes may be caused by *Bacteroides*, *Bifidobacterium*, and *Feacalibacterium prausnitzii* fermenting citrus peel pectin for growth.

In this study, CMPP can be considered as pectin due to being mainly composed of Gal A, Gal, Ara and Rha, and showed immunological activity and modulated gastrointestinal metabolic function. Current research has shown that pectin is a soluble dietary fiber and has health promoting properties through modulating the risks of disease, such as type 2 diabetes, obesity, cardiovascular diseases, colorectal cancer, inflammation and immunodeficiency. It can involve microbiota-independent and microbiota-dependent factors. As for microbiota-independent factors, immunological activity is related to the structural characteristics of pectin. Combined with previous study, CMPP contained structural fragments of CMPP-1 and CMPP-2, which was the major reason for similar immunological activity with increasing secretion of cytokines (NO, TNF-α and IL-6). However, the extraction process of CMPP was simpler than that of CMPP-1 and CMPP-2, and more yield can be obtained for research. In terms of microbiota-independent factors, the impact of pectin on intestinal health derives from its fermentation into SCFAs, which are predominantly associated with immune regulation and prevention of inflammation [50]. SCFAs also serve as the source of energy for gut microbes to improve microbial composition. It is reported that the foods rich in dietary fiber can increase the relative abundance of certain bacteria (e.g., *Bifidobacterium*, *Lactobacillus*, *Enterococcus* and *Ruminococcus*), increasing acetic acids and butyric acids in the proximal colon [14]. The Goji polysaccharides could remarkably promote the growth of *Clostridium cluster*, *F. prausnitzii* and and *Roseburia* spp., thereby notably increasing the content of butyrate and alleviating IL-10 deficient colitis in mice [51]. *Lycium barbarum* oligosaccharides promoted the growth of beneficial bacteria, including *Akkermansia*, *Bacteroides*, *Lactobacillus* and *Prevotella*, and reduced the symptoms of inflammation, insulin resistance and hyperglycemia in the T2D mouse model [52]. CMPP were fermented to increase acetic acid, propionic acid and butyric acid during the intake period, stimulating the growth of *Akkermansia*, *Bacteroides*, *Bifidobacterium*, *Feacalibacterium*, and *Roseburia*. The metabolism effects of these gut bacteria could be considered to regulate T2D, obesity and inflammation. Moreover, the cross-feeding interaction of gut symbionts is also important for metabolism. For example, *Bacteroides* spp. provide a cross-feeding of dextran to probiotics, such as *Bifidobacterium* spp. [53]. CMPP might also regulate composition of gut microbiota and metabolic in this way. Differences in the composition of the gut microbiomes would influence regulation of metabolism, immunity and disease. In order to supplement more information on human health, it is worth studying the potential effects of different pectin for developing its selective regulation [54].

## 4. Conclusions

As a pectin polysaccharide, CMPP is rich in glucuronic acid, arabinose, galactose and rhamnose, and its biological activities are probably related to its composition. As described in this study, CMPP could contribute to immunity enhancement by promoting the cell proliferation and increasing the release of cytokines (NO, TNF-α and IL-6) on RAW264.7 macrophages. On the other hand, CMPP could be fermented into SCFAs to modulate the growth of gut bacteria, including *Akkermansia*, *Bacteroides*, *Bifidobacterium*, *Feacalibacterium*, and *Roseburia*, decreasing the ammonia concentration. These metabolism effects could be considered to regulate T2D, obesity and inflammation. Taken together, the results showed that CMPP had potential immunological activity and prebiotic effects.

To better understand the health effects of CMPP, the following issues remain to be further investigated: (1) To determinate the appropriate range concentration of CMPP and intervention duration that benefit health. Identification of CMPP as a prebiotic and determination of the appropriate doses is challenging, but helping. (2) To understand the complex influence of CMPP on gut microbiota and related diseases in vivo, and based on functional properties of gut microbiomes, to establish a concerning database for personalized nutrition management. (3) To decipher the impact of processing methods on CMPP, developing the recycling of fruit and vegetable by-products in the food industry. These investigations will be crucial for the relationships among pectin, gut microbiota, and host health.

## Figures and Tables

**Table 1:** Average values of the qPCR counts (log copy number/mL) for bacterial group analysis during gastrointestinal digestion of CMPP. (* was significantly different, *p* < 0.05, vs. the stabilization period).

Bacterial Group	Compartment	Stabilization Period	Intake Period	Washout Period
Total bacteria	AC	9.41 ± 0.18	9.44 ± 0.02	9.47 ± 0.08
	TC	9.29 ± 0.21	9.36 ± 0.17	9.22 ± 0.14
	DC	9.14 ± 0.11	9.27 ± 0.08	9.23 ± 0.03
*Akkermansia*	AC	-	-	-
	TC	8.33 ± 0.50	9.06 ± 0.05	8.75 ± 0.20
	DC	8.58 ± 0.37	8.82 ± 0.01	8.91 ± 0.06
*Bacteroides*	AC	9.63 ± 0.09	9.94 ± 0.15 *	9.90 ± 0.06 *
	TC	9.64 ± 0.11	9.78 ± 0.15	9.52 ± 0.25
	DC	9.26 ± 0.34	9.39 ± 0.15	9.55 ± 0.23
*Bifidobacterium*	AC	8.55 ± 0.12	8.64 ± 0.06	8.54 ± 0.16
	TC	8.39 ± 0.37	8.42 ± 0.14	8.05 ± 0.23
	DC	8.18 ± 0.28	8.35 ± 0.09	8.26 ± 0.29
*Feacalibacterium*	AC	4.01 ± 0.49	4.63± 0.10	4.45 ± 0.03
	TC	4.66 ± 0.28	4.82 ± 0.09	4.64 ± 0.31
	DC	4.34 ± 0.20	4.83 ± 0.04 *	4.76 ± 0.14 *
*Roseburia*	AC	5.53 ± 0.10	6.35 ± 0.13 *	5.69 ± 0.10
	TC	5.51 ± 0.41	5.98 ± 0.12	5.41 ± 0.20
	DC	5.47 ± 0.34	5.99 ± 0.27	5.63 ± 0.05

## Data Availability

Data is contained within the article.

## Abbreviations

AC	ascending colon
Ara	arabinose
BFBL	functional biology of lactic bacteria
CMPP	*Cucumis metuliferus* peels polysaccharide
CMPP-1	fraction 1 of CMPP
CMPP-2	fraction 2 of CMPP
DC	descending colon
DMEM	Dulbecco modified Eagle’s medium
DMSO	dimethyl sulfoxide
ELISA	enzyme-linked immunosorbent assay
FBS	fetal bovine serum
Fuc	fucose
Gal	galacturonic
Gal A	galacturonic acid
Glu	glucose
Glu A	glucose acid
HPAEC-PAD	high-performance anion-exchange chromatography with pulsed amperometric detection
HPLC	high efficiency liquid chromatography
IL-6	interleukin 6
IL-10	interleukin 10
LPS	lipopolysaccharide
Man	mannose
Man A	mannose acid
NO	nitric oxide
qPCR	quantitative real-time polymerase chain reaction
RAW264.7	mouse mononuclear macrophage leukemia cells
Rha	rhamnose
SCFAs	short-chain fatty acids
T2D	type 2 diabetes
TC	transverse colon
TFA	trifluoracetic acid
TNF-α	tumor necrosis factor α
UV	ultraviolet and visible
Xyl	xylose
